# Heterogeneity and Utility of Pharmaceutical Company Sharing of Individual-Participant Data Packages

**DOI:** 10.1001/jamaoncol.2023.3996

**Published:** 2023-10-05

**Authors:** Ashley M. Hopkins, Natansh D. Modi, Ahmad Y. Abuhelwa, Ganessan Kichenadasse, Nicole M. Kuderer, Gary H. Lyman, Michael D. Wiese, Ross A. McKinnon, Frank W. Rockhold, Aaron Mann, Andrew Rowland, Michael J. Sorich

**Affiliations:** 1 College of Medicine and Public Health, Flinders University, Adelaide, South Australia, Australia; 2 College of Pharmacy, University of Sharjah, Sharjah, United Arab Emirates; 3 Flinders Centre for Innovation in Cancer, Department of Medical Oncology, Flinders Medical Centre, Adelaide, South Australia, Australia; 4 Advanced Cancer Research Group, Seattle, Washington; 5 Public Health Sciences Division, Fred Hutchinson Cancer Research Center, Seattle, Washington; 6 Clinical & Health Sciences, University of South Australia, Adelaide, South Australia, Australia; 7 Department of Biostatistics and Bioinformatics, Duke University, Durham, North Carolina; 8 Clinical Research Data Sharing Alliance, Piscataway, New Jersey

## Abstract

**Importance:**

The pharmaceutical industry has made substantial investments in developing processes for sharing individual-participant data (IPD) from clinical trials. However, the utility and completeness of shared IPD and supporting documents must be evaluated to ensure the potential for scientific advancements from the data sharing ecosystem can be realized.

**Objective:**

To assess the utility and completeness of IPD and supporting documents provided from industry-sponsored clinical trials.

**Design, Setting, and Participants:**

From February 9, 2022, to February 9, 2023, 91 of 203 clinical trials supporting US Food and Drug Administration registrations of anticancer medicines for the treatment of solid tumors from the past decade were confirmed as eligible for IPD request. This quality improvement study performed a retrospective audit of the utility and completeness of the IPD and supporting documents provided from the 91 clinical trials for a planned meta-analysis.

**Exposures:**

Request for IPD from 91 clinical oncology trials indicated as eligible for the request.

**Main Outcomes and Measures:**

The utility and completeness of the IPD and supporting documents provided.

**Results:**

The IPD packages were obtained from 70 of 91 requested clinical trials (77%). The median time to data provision was 123 (range, 117-352) days. Redactions were observed in 18 of the acquired IPD packages (26%) for outcome data, 11 (16%) for assessment variables, and 19 (27%) for adjustment data. Additionally, 20 IPD packages (29%) lacked a clinical study report, 4 (6%) had incomplete or missing data dictionaries, and 20 (29%) were missing anonymization or redaction description files. Access to IPD from 21 eligible trials (23%) was not granted.

**Conclusions and Relevance:**

In this quality improvement study, there was substantial variability within the provided IPD packages regarding the completeness of key data variables and supporting documents. To improve the data sharing ecosystem, key areas for enhancement include (1) ensuring that clinical trials are eligible for IPD sharing, (2) making eligible IPD transparently accessible, and (3) ensuring that IPD packages meet a standard of utility and completeness.

## Key Points

**Question:**

What is the utility and completeness of individual-participant data (IPD) and supporting documents provided from industry-sponsored clinical trials through data transparency processes?

**Findings:**

In this quality improvement study, IPD packages were received from 70 of 91 eligible clinical oncology trials (77%) for a planned meta-analysis. In addition to access being denied for 21 of the eligible trials (23%), there was substantial variation in the completeness of key data variables and supporting documents across the provided IPD packages.

**Meaning:**

These findings suggest that there is a substantial opportunity to enhance the data sharing ecosystem, including ensuring that clinical trials indicated as eligible for sharing are truly accessible and that IPD packages meet a standard of utility.

## Teaser

This quality improvement study evaluates the utility of data provided by pharmaceutical companies for a planned meta-analysis of adverse events and therapeutic outcomes in trials of recently registered anticancer medicines using individual-participant data.

## Introduction

**Figure 1.**
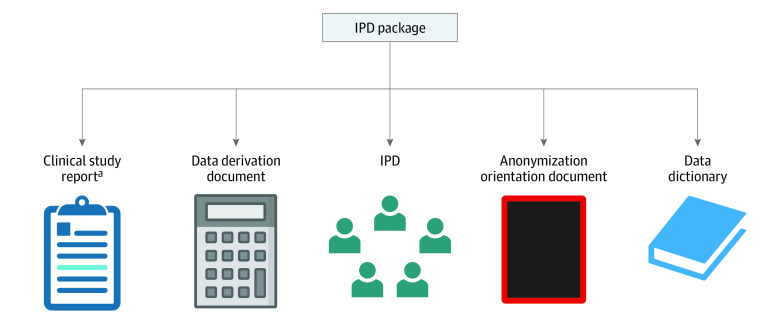
Example of Key Components Within an Individual-Participant Data (IPD) Package to Facilitate Independent Research Created using BioRender Premium scientific and illustration software.^4^ ^a^Includes detailed information on the study protocol, statistical analysis plan, and results.

Over the past decade, pharmaceutical companies have implemented data sharing policies to enable independent researchers to access individual-participant data (IPD) packages (Figure 1) from clinical trials.^1,2,3^ Sharing IPD allows verification of results, generation of hypotheses, and the undertaking of analyses that can inform clinical practice and improve trial designs.^3,5,6,7,8^ To achieve these outcomes, the data sharing ecosystem must operate efficiently and have redaction and anonymization processes that maintain IPD usability as well as preserve participant confidentiality.^8,9,10,11,12,13,14^ Toward this end, substantial efforts have gone into developing processes to facilitate data requests and infrastructures to anonymize and protect IPD.^3,15^ In addition, the significance of providing adequate supporting documents, such as clinical study reports (CSRs), data dictionaries, data derivation, and anonymization orientation documents, has been emphasized to ensure researchers can understand the IPD when it is provided.^9,13,16^

Presently, no US or European Union regulations mandate IPD sharing from industry-sponsored medicine trials. Most pharmaceutical companies have independently developed their own data sharing processes,^1^ which potentially leads to heterogeneity in IPD anonymization and redaction processes and supporting documentation provisions between companies. This study aimed to evaluate the utility of data provided by pharmaceutical companies for a planned IPD meta-analysis of adverse events and therapeutic outcomes for recently registered anticancer medicines. By exploring how different sponsors provide IPD and supporting documents to independent researchers, we hope to contribute to developing strategies that improve IPD sharing practices.^3,11,15^

## Methods

### Sample

In this quality improvement study, an audit conducted from February 9, 2022, to February 9, 2023, confirmed that 91 of 203 clinical trials supporting US Food and Drug Administration (FDA) registrations of anticancer medicines against solid tumors from the past decade were eligible for independent IPD request.^17^ On February 9 and 10, 2022, IPD requests were submitted to the appropriate data sponsors outlining a planned meta-analysis summarizing adverse events and therapeutic outcomes by race and sex from these eligible trials. The eMethods in Supplement 1 details the submitted research proposal.

For each trial, we had information on the National Clinical Trial number, data sponsor, investigated medicine, trial phase, primary completion date, cancer type, and IPD request details (eg, whether the request process was internal or facilitated by Vivli,^18^ ClinicalStudyDataRequest.com,^19^ or Yale University Open Data Access).^20^ An exploratory investigation of the Project Data Sphere portal^21^ was also conducted to assess whether any of the sampled trial IPD were available on that platform.

### Key Data and Supporting Documentation Provisions

For the clinical trials for which IPD packages were shared, we report the heterogeneity in the provision of key outcome (adverse events, progression-free and/or disease-free survival, and overall survival), assessment variable (race, ethnicity, and sex), and adjustment (baseline age, weight, and performance status) data for the described IPD meta-analysis. Heterogeneity was evaluated according to whether key variables were fully redacted or partially redacted (eg, removing data entries for some participants, or categorizing the variable to cause a loss of information). We also report whether CSRs, data dictionaries, data derivation, and anonymization orientation documents were provided. Before recording key variables or supporting documentation as redacted or missing within an IPD package, the study team requested the variable or document from the data sponsor.

### Statistical Analysis

This study reports the success rate and time to receipt for obtaining IPD packages. The findings are reported after 12 months of follow-up, completed on February 9, 2023. Reasons for nonprovision of trial IPD were documented. Potential differences in IPD provision rates according to primary completion dates and trial phases were assessed via χ^2^ tests with Yates continuity correction. Analysis was performed using R, version 4.1.0 (R Project for Statistical Computing). One-sided *P* < .05 indicated statistical significance.

## Results

### Sample

**Figure 2.**
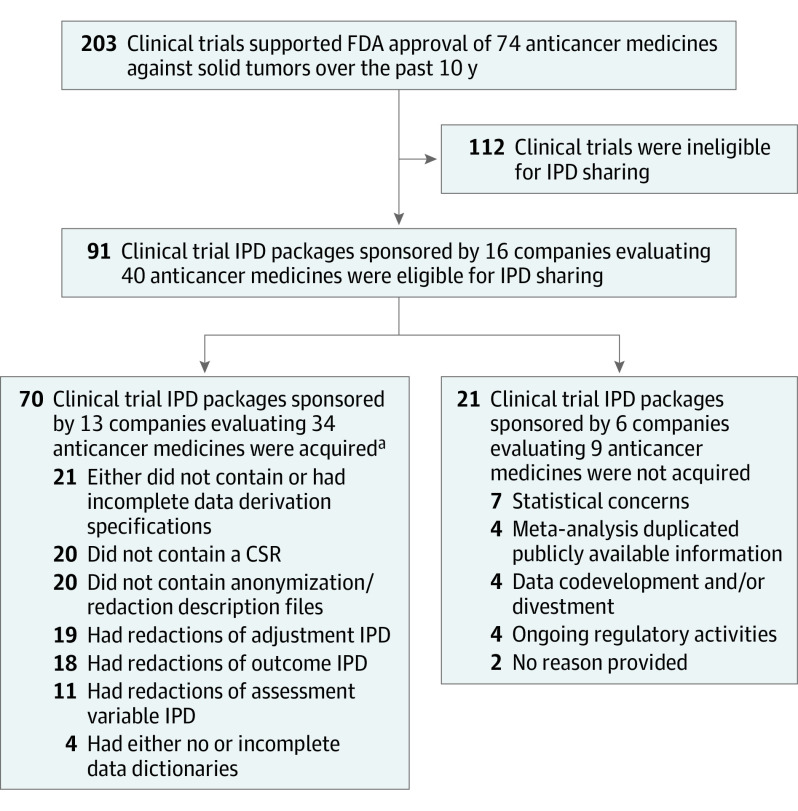
Flow Diagram of the Individual-Participant Data (IPD) and Supporting Documents Requested and Accessed in This Study CSR indicates clinical study report; FDA, US Food and Drug Administration. ^a^Includes data from more than 45 000 participants. The median time from IPD request to IPD access was 123 days (range, 117-352 days).

We requested IPD packages from 91 clinical trials sponsored by 16 pharmaceutical companies, including 15 within the top 50 pharmaceutical companies by global revenue for 2021.^22^ These trials supported the FDA registration of 40 anticancer medicines against solid tumors in the past 10 years, including trials evaluating antibody-drug conjugates, cytotoxic agents, hormonal therapies, immunomodulatory drugs, and noncytotoxic targeted drugs. Figure 2 presents a flow diagram of the IPD and supporting documents that were requested and accessed; the eAppendix in Supplement 1 contains the raw data that were collected.

### Data Provision Success Rate and Timing

We obtained IPD packages from 70 of the 91 clinical trials (77%). This included data from 13 companies to form a pooled cohort of more than 45 000 patients treated with 34 contemporary anticancer medicines. The median time to IPD package provision was 123 days (range, 117-352 days).

For 21 of 91 requested clinical trials (23%), we were not provided IPD packages. This includes data from 6 companies, 9 anticancer medicines, and approximately 12 000 trial participants. The reasons for not providing IPD packages for these clinical trials were not given (n = 2), ongoing regulatory activities (n = 4), data codevelopment and/or divestment (n = 4), meta-analysis repetitive with publicly available information (n = 4), and concerns with the planned statistical evaluations (n = 7). For the 11 clinical trials for which IPD were not shared due to meta-analysis being repetitive with publicly available information or statistical concerns, the company made these decisions internally rather than through an independent review panel assigned to assess the submitted research proposal.

The success rate of acquiring clinical trial IPD was not associated with either time since primary completion or trial phase. Specifically, as of February 9, 2022, it had been less than 5 years since primary completion for 21 of the 70 clinical trials that were shared (30%) and 9 of the 21 clinical trials that were not shared (43%) (*P* = .38). Furthermore, 53 of the 70 shared clinical trials (76%) were phase 3, and 14 of the 21 clinical trials that were not shared (67%) were phase 3 (*P* = .59). An exploratory investigation of the Project Data Sphere platform identified only 2 control groups of the 91 sampled trials as available for download.

### Redaction of Key Data Variables

Of the 70 clinical trials with provided IPD packages, 18 (26%) had redactions of outcome data, 11 (16%) had assessment variable redactions, and 19 (27%) had adjustment data redactions that could complicate planned analyses. In terms of outcomes, 6 IPD packages (9%) did not contain overall survival data, 6 (9%) did not have progression-free and/or disease-free survival data, and 12 (17%) had adverse event data partially redacted (removing either rare adverse event information or the Medical Dictionary for Regulatory Activities [MedDRA]-preferred term). Race IPD were completely removed from 5 packages (7%) and partially redacted from another 5 (7%). From 1 trial (1%), sex IPD were removed. Concerning key adjustment variables, age was partially redacted in 18 IPD packages (26%), weight was removed from 10 (14%), and performance status information was removed from 2 (3%).

Although substantial redaction heterogeneity was observed, it was estimated that all provided IPD packages would contribute to at least 1 domain within the submitted research proposal. Notably, from 5 companies, we requested IPD from more than 5 clinical trials. Two of these companies provided access to 100% of the trials requested of them, without redaction to any of the key outcomes, assessment variables, or adjustment data.

### Supporting Documentation

Of the 70 clinical trials with provided IPD packages, 20 (29%) did not contain a CSR, 4 (6%) had no or incomplete data dictionaries, 21 (30%) had no or incomplete data derivation specifications, and 20 (29%) lacked anonymization or redaction guides. Further, data derivation and anonymization guides had substantial structural variabilities between packages. The data derivation guides ranged from brief notes on selected variables to detailed information cross-referencing all metadata specifications between files. Anonymization guides ranged from brief notes to detailed information on data anonymization processes and variable availability across data cuts (important as overall survival and progression-free survival are often available in different data cuts). Of the 13 companies providing IPD packages to this study, 4 routinely provided CSR, data dictionary, data derivation, and anonymization orientation documents within their IPD packages.

## Discussion

From a sample of 91 industry-sponsored clinical oncology trials confirmed eligible for sharing, IPD was successfully acquired from 70 (77%) for a planned meta-analysis. In these IPD packages, 18 (26%) had redactions of outcome data, 11 (16%) had assessment variable redactions, and 19 (27%) had adjustment data redactions that could complicate planned analyses. Further, 20 (29%) lacked a CSR, 4 (6%) had no or incomplete data dictionaries, 21 (30%) had no or incomplete data derivation specifications, and 20 (29%) lacked anonymization or redaction guides. Overall, this study demonstrates considerable room for improvement in IPD package provision practices.

Recent research evaluated the ability to retrieve IPD from a sample of clinical trials for Alzheimer disease and diabetes medications.^10^ The sample included both industry- and non–industry-sponsored trials, with no restrictions on trial age.^10^ Their sample of 108 trials included 11 publicly funded trials and 19 trials for which the authors could not establish funding information. The IPD could not be accessed from any of these 30 randomized clinical trials.^10^ For the remaining 78 industry-sponsored trials, IPD could only be accessed from 26 (33%).^10^ Of the 52 industry-sponsored trials where IPD could not be accessed, 40 (77%) were noted as either too old with IPD destroyed, or there was difficulty establishing IPD ownership.^10^ Conversely, our study focused on obtaining IPD from 91 contemporary industry-sponsored oncology trials recently confirmed with the data sponsor as eligible for IPD request. In this study, we accessed IPD packages from 77% (70 of 91) of the requested trials, resulting in a sample size able to give detailed information on the utility of IPD and supporting documentation provided by data sponsors for planned meta-analyses.

At present, accessing high-quality IPD from non–industry-sponsored trials is extremely difficult and often impossible.^10,12,14,23,24,25,26^ As such, this study focused on the acquisition and utility of IPD packages from industry-sponsored oncology trials for recently approved anticancer medicines. For many newer medicines, these clinical trials are the centerpiece of efficacy and safety; thus, their IPD represent a key resource for enriching the postapproval evidence base. This study was designed to gain a comprehensive understanding of industry heterogeneity in IPD and supporting documentation provisions. By understanding that, efforts toward harmonizing IPD sharing practices between companies and developing an optimized data sharing ecosystem can be made. Over time, optimized processes may then permeate throughout academia and pharmaceutical companies that do not consistently share IPD.

In this study, we obtained IPD from 70 clinical trials, totaling a pooled cohort of more than 45 000 patients receiving contemporary anticancer medicines against solid tumors. Within the sample, 5 companies had more than 5 clinical trials requested. Notably, 2 of these companies provided access to 100% of the trials requested of them, without redaction of any key IPD required for the approved proposal. Their provisions likely resulted from (1) clear processes to confirm trial-sharing eligibility, (2) reliance on independent scientific review panels for proposal reviews, and (3) data protection methods commensurate to facilitate research.^27,28^ Conversely, redaction or nonprovision of key IPD variables likely results from company processes that limit access to multiple data cuts or the implementation of overly strict IPD protection methods. For example, we propose that the appropriateness of redacting rare adverse event data or MedDRA-preferred terms from IPD packages requires evaluation. Furthermore, 11 of the 21 clinical trials were inaccessible due to internal company decisions that the meta-analysis was repetitive with publicly available information and statistical concerns. Arguably these decisions should have been made by an independent review panel.

In addition to IPD utility, we evaluated the provision of supporting documents within IPD packages. Supporting documents such as CSRs, data dictionaries, data derivation, and anonymization guides are crucial for researchers to understand provided data and to check the accuracy of their standardization processes.^3,11,29,30^ Such checks are critical safeguards to ensuring the validity of the data sharing ecosystem.^3,11,29,30^ Of the 13 companies that provided IPD packages, only 4 routinely included a CSR, data dictionary, data derivation, and/or an anonymization guide. To enhance the utility of shared IPD, companies must provide these key supporting documents, and the documents should be of high quality.

An exploratory investigation of Project Data Sphere identified IPD packages available for download from 2 of the 91 clinical trials in our sample. Project Data Sphere is an oncology-focused platform, unique in enabling open access to IPD by qualified researchers.^31^ The platform is supported by 14 pharmaceutical industry contributors who, when they contribute, most often provide IPD from the control group of clinical trials.^31^ Notably, open access models enable IPD downloads to local computers, which lessens statistical restrictions and more easily enables crowd-sourced research.^32^ Through this approach, Project Data Sphere has facilitated multiple collaborative works in top medical journals that may not have been possible with other access arrangements.^33^ Given the importance of the sampled trials toward recent anticancer medicine registrations, it was concerning that only data from 2 of the trials were accessible on the platform.

### Strengths and Limitations

To our knowledge, this study represents the first systematic evaluation of the utility of IPD provided for a planned meta-analysis of oncology trials leveraged via data sharing policies of the pharmaceutical industry. The data contributors evaluated represent some of the largest pharmaceutical companies in the world. We acknowledge that the ability to use IPD from smaller companies may differ and that the research proposal may have influenced companies’ decisions to share IPD. Nonetheless, this study demonstrates that the IPD sharing practices of pharmaceutical companies urgently require harmonization. Key considerations include clear independent evaluations of research proposals, IPD anonymization and redaction processes that protect data utility, and comprehensive provision of supporting documentation. These measures are required to increase the utility of shared IPD so that the data sharing ecosystem can achieve its goals.

This study also has some limitations. It is critical to acknowledge that while the *Cochrane Handbook for Systematic Reviews of Interventions* concedes that the IPD approach has substantial potential to improve medicine understandings, the realization of the vision is affected by issues of IPD utility and availability.^34^ We initially identified 203 clinical trials that had results presented in the product labels of anticancer medicines against solid tumors registered by the FDA over the past decade; of those 203 trials, 91 were indicated to our team as eligible for sharing, and of those, we acquired IPD from 70. This corresponds to 34% of the original 203 oncology trials being accessed. Thus, while this study focused on ensuring that the IPD packages that were provided were of high utility, it remains a crucial issue to ensure that IPD of key trials are available and shared. Vitally, where there is no undue risk of participant reidentification, all IPD from clinical trials that support medicine registrations should be independently accessible.^17,35,36,37^ This is a goal that should be achieved immediately, rather than in the distant future.^35^

## Conclusions

In this quality improvement study, we acquired IPD packages from 70 of 91 clinical trials (77%) supporting FDA-approved anticancer medicines against solid tumors from the past decade. However, access to IPD was denied for 21 of the trials (23%). Furthermore, we noted substantial variability in the completeness of key data variables and supporting documents across the provided IPD packages. We identified the following key calls to action to optimize the data sharing ecosystem: (1) clinical trial IPD should be eligible for sharing, (2) IPD should be transparently accessible with request processes fully mediated through independent processes, and (3) provided IPD packages should meet a high standard of utility and completeness. It is noteworthy that several major pharmaceutical companies already aim to achieve these benchmarks, serving as evidence of feasibility. If these transparency practices are adopted more widely, an improved data sharing ecosystem can be cultivated that ultimately drives an ability to deliver the best possible information about medicines to patients. Furthermore, those espousing these practices should be held accountable for their performance.

## Notes

Supplement 1. **eMethods.** Research Proposal
eReferences
**eAppendix.** Raw Data

Supplement 2.
Data Sharing Statement
