# Supplementary material for: Heterogeneity and Utility of Pharmaceutical Company Sharing of Individual-Participant Data Packages
Source: JAMA Oncol. 2023 Oct 5;9(12):1621–6. doi: 10.1001/jamaoncol.2023.3996 (PMC10557028; doi:10.1001/jamaoncol.2023.3996)
Supplement: Supplement 1. — eMethods. Research Proposal eReferences eAppendix. Raw Data [file jamaoncol-e233996-s001.pdf]

Supplementary Online Content

Hopkins AM, Modi ND, Abuhelwa AY, et al. Heterogeneity and utility of pharmaceutical company sharing of individual-participant data packages. *JAMA Oncol*. Published online October 5, 2023. doi:10.1001/jamaoncol.2023.3996

eMethods. Research Proposal

eReferences

eAppendix. Raw Data

This supplementary material has been provided by the authors to give readers additional information about their work.

© 2023 Hopkins AM et al. *JAMA Oncology*.

eMethods. Research Proposal

Summarising the therapeutic and adverse effects of anticancer medicines according to race and sex: a pooled analysis of clinical trials of contemporary treatments for solid tumours.

Research Proposal

Narrative summary explaining the relevance of the project to science and public health.

Over the last decade there has been substantial advancement in the treatment of solid tumours (e.g., lung, breast, colorectal, prostate cancer), including the introduction of immune checkpoint inhibitors (a type of anticancer medicines), targeted therapies, and novel chemotherapies. However, response and toxicity to many of these medicines remains highly unpredictable. Two factors urgently requiring investigation are potential differences in therapeutic and adverse effects of contemporary anticancer medicines according to race and sex [1, 2].

Race differences are associated with a significant health disparity gap. For many common malignancies there are substantial differences in incidences according to race, while race is a phenotype for differences in genetic and tumour biology factors. Further there are inequities in drug development processes (e.g., in reporting and representation) and it is unclear if this is resulting in systematic disparities in anticancer treatment therapeutic and adverse effects [1, 3-10]. Science is also becoming increasingly aware that sex is an important modifier of health, disease and medicine efficacy [11]; however, the availability of quality information to inform sex (or gender) differences in outcomes from anticancer medicines are currently limited [2, 12]. This project will bring together individual participant data from key clinical trials to summarise the therapeutic and adverse effects of contemporary anticancer medicines according to race and sex. Being able to appraise the expected response and adverse effect profile of anticancer treatments according to racial and sex differences will enable patients and clinicians to make better patient-centred decisions.

Specifically, this project will endeavour to gather and then utilise data available via pharmaceutical company data sharing policies to summarise the therapeutic and adverse effects of contemporary anticancer medicines according to race and sex.

Aims/Objectives and Hypotheses

The hypothesis of this research is that if the pharmaceutical industry has functioning and timely data transparency policy, then it will be leverageable to pro vide information summarising the therapeutic and adverse effects of contemporary anticancer medicines according to race and sex.

Specifically, this study aims to:

1. Summarise the key adverse effects of anticancer medicines registered by the FDA in the past decade according to race and sex.
2. Summarise the key therapeutic outcomes of anticancer medicine registered by the FDA in the past decade according to race and sex.

Purpose of Analysis

Participant-level data meta-analysis

© 2023 Hopkins AM et al. *JAMA Oncology*.

Study Design

Brief Description

Our research team has systematically collated information on anticancer medicines registered by the United States, Food and Drug Administration (FDA) in the past decade for the treatment of solid tumours. The key clinical trials that backbone the registration of these medicines was then collated from the product information sheets prepared by the sponsoring pharmaceutical company. It was then confirmed with the sponsoring pharmaceutical company that sharing of individual participant data from these clinical trials would be considered upon receipt of a valid research proposal. This study will endeavour to gather and pool individual participant data from phase 2 and 3 clinical trials which have been indicated to the team as within scope for sharing and would be considered for sharing upon receipt of a full proposal, with the purpose of this project to systematically characterize the adverse event and therapeutic response profiles of contemporary treatment options for solid tumours according to race and sex. Analyses will utilise per-protocol populations and all study arms shared. This study will utilise a 2-stage individual-participant data meta-analysis approach. As a perspective to the scientific community, a summary of trial sponsors willing and not willing to participate individual participant data to this study will also be published (noting all data requested in the study has previously been indicated as within scope for sharing and would be considered for sharing upon receipt of a full proposal).

Outcome Elements Categorization/Definitions

Individual participant data are required for adverse event and therapeutic outcomes including clinician/patient reported adverse effects according to grade or sentinel events, and response, progression-free survival/ disease-free survival, and where available overall survival. Event flag, censoring and time after treatment initiation data will be calculated for these outcomes.

Main Predictor / Independent Variable

The coprimary predictors of adverse event and therapeutic outcomes to be evaluated in this study will be race and sex. Race is commonly collected within clinical oncology trials, often categorised within individual participant data as white, Asian, black, and other. Sex data is most commonly collected within clinical oncology trials as male versus female. This study will provide summarisation of race, sex and gender data which has been recorded during trial execution.

Other Variables of Interest

Where available summaries of available ethnicity, socioeconomic, geographical region, patient reported financial statuses, patient-reported outcomes, concomitant medicine, and medical history data according to race and sex will be reported. Exploratory analyses adjusted for age, weight, performance status, cancer type, stage of disease, and line of therapy will be conducted – missing data will be summarised. Individual participant data on the above outcome, covariate and adjustment data will be required.

Publication Plans

A summary of the proposed research plan will be posted publicly immediately following acceptance of the research proposal. Results of all completed analyses will be published in peer-reviewed international publications and where possible presented at scientific meetings. Manuscript(s) will be targeted primarily to international oncology journals (e.g., British Journal of Cancer, International Journal of Cancer) and will be submitted as soon as possible following completion of the requisite analyses.

© 2023 Hopkins AM et al. *JAMA Oncology*.

As a related perspective piece to the scientific community (independent on the planned meta-analyses): At 9-months from initial proposal submission, summaries of trial sponsors not willing (or unable) to participate individual participant data to this study will collated with the aim to publish at 12-months (noting all data requested in the study has previously been indicated as within scope for sharing and would be considered for sharing upon receipt of a full proposal). The time from proposal submission to data access will be included in the publication, as well as a summary of the scope of data (IPD and supporting documentation) provided by each sponsor.

General Statistical Analysis Plan

Population:

The research team has systematically collated information on anticancer medicines registered by the FDA in the past decade for the treatment of solid tumours. The key clinical trials which backbone the registration of these medicines was then collated from the product information sheets prepared by the sponsoring pharmaceutical company. It was then confirmed with the sponsoring pharmaceutical company that sharing of individual participant data from these clinical trials would be considered upon receipt of a valid research proposal. This study will pool individual participant data from phase 2 and 3 clinical trials to characterize the adverse event and therapeutic response profiles of contemporary treatment options for solid tumours according to race and sex (noting it was previously confirmed that proposals for access to the individual participant data from these requested trials would be considered upon receipt of a valid research proposal). Analyses will utilise per-protocol populations and all study arms shared. Information on clinical trials which are not shared by the industry sponsor will be highlighted prior to analysis.

Primary and Secondary Endpoints:

The primary aims of the study are to:

1. Summarise the key adverse effects of anticancer medicine registered by the FDA in the past decade according to race and sex. Data are required on clinician/patient reported adverse effects according to grade or sentinel events.
2. Summarise the key therapeutic outcomes of anticancer medicine registered by the FDA in the past decade according to race and sex. Data are required for progression-free survival/ disease-free survival, and where available overall survival.

Event flag, censoring and time after treatment initiation data will be calculated for these endpoints.

Primary predictor and sensitivity analyses:

The coprimary predictors of adverse event and therapeutic outcomes to be evaluated in this study will be race and sex. Race is commonly collected within clinical oncology trials, often categorised within individual participant data as white, Asian, black, and other. Sex data is most commonly within clinical oncology trials as male versus female. This study will provide summarisation of race, sex and gender data which has been recorded during trial execution. Where available summaries of available ethnicity, socioeconomic, geographical region, patient reported financial statuses, patient-reported outcomes, concomitant medicine, and medical history data according to race and sex will be reported. Exploratory analyses adjusted for age, weight, performance status, cancer type, stage of disease, and line of therapy will be conducted – missing data will be summarised.

Software:

The R Software (R Core Team) will be used for data preparation, modelling and graphical output.

© 2023 Hopkins AM et al. *JAMA Oncology*.

Statistical analysis:
Crude associations for adverse events will be reported based cohort frequencies of treatment induced grade ≥ 1, grade ≥ 3, and sentinel (e.g., drug cessation, hospitalisation, or death) adverse events according to race and sex. Crude associations for therapeutic outcomes will be reported based upon Kaplan Meier estimates of median time to events (e.g., median time to progression-free survival, disease-free survival, and overall survival) according to race and sex.

This study will utilise a 2-stage individual-participant data meta-analysis approach (i.e., as per standard meta-analysis techniques, each trial and arm will be analysed independently prior to pooling). Cox proportional hazard analysis will be used to assess the association between race/sex and the time to adverse events/ survival time (estimated in the first instance individually for each trial arm). Associations will be reported as hazard ratios (HRs) with 95% confidence intervals (CI). HRs and 95%CI will then be pooled and presented in forest plot according to conventional meta-analysis techniques. Heterogeneity between trials, drug class, and companies will be evaluated via the I^2 statistic (i.e., evaluating the appropriateness of presenting pooled findings). P-values <0.05 will be considered statistically significant. Analyses based upon therapeutic classes will also be conducted to enable a better understanding of whether the relationships identified are specific to certain classes of treatment or are common to contemporary treatment options. As this analysis is primarily hypothesis generating and will require subsequent validation of any findings, no formal adjustment for multiple testing is intended. However, this limitation will be clearly stated in any publications of results. As it is expected that < 5% of race and sex data will be missing, a complete case analysis is planned. Exploratory analyses adjusted for available age, weight, performance status, cancer type, stage of disease, and line of therapy data will be conducted. Summary statistics of available ethnicity, socioeconomic, geographical region, patient reported financial statuses, patient-reported outcomes, concomitant medicine, and medical history data according to race and sex will also be reported.

Power:
Whether race and sex are predictors of substantial (e.g., double the risk) differences in mortality and adverse effects will be of primary interest. Based upon a 30% incidence of toxicity, a sample size of approximately 600 is required to detect a predictor (with a 10% frequency within the population) associated with a two-fold risk (α=0.05 with 80% power). Based upon an event rate of 40% during trial follow-up (e.g. for progression), approximately 450 participants are required for 80% power to detect a predictor (with a 10% frequency within the population) associated with a two-fold hazard of the event (α=0.05). These samples sizes are well within scope for this study. Sample sizes greater than this will allow exploratory adjusted analyses.

Quality Control:
Data will be explored for inconsistencies in time recordings, physiologically unreasonable covariate values, and unit errors. Prior to beginning analyses, individual data values will be extracted/constructed based on the raw and analysis datasets provided. To ensure that each predictor and outcome variable has been correctly extracted/constructed from the data provided, basic analyses and descriptive statistics will be reproduced to check for consistency with pertinent results in published manuscripts or clinical study reports relating to the specific trial. Where there are insufficient published results to confirm the proper extraction of the variable, the extracted values will be manually checked against a random sample of the original dataset values.

Countries where analysis will be conducted
Australia

© 2023 Hopkins AM et al. *JAMA Oncology*.

eReferences

1. Stein, J.N., M. Charlot, and S. Cykert, *Building Toward Antiracist Cancer Research and Practice: The Case of Precision Medicine.* JCO Oncol Pract, 2021. **17**(5): p. 273-277.
2. Oncology, u.S.f.M. *ESMO GENDER MEDICINE TASK FORCE*. 2021; Available from: https://www.esmo.org/about-esmo/organisational-structure/esmo-task-forces/esmo-gender-medicine-task-force.
3. Loree, J.M., et al., *Disparity of Race Reporting and Representation in Clinical Trials Leading to Cancer Drug Approvals From 2008 to 2018.* JAMA Oncology, 2019. **5**(10): p. e191870-e191870.
4. Ajewole, V.B., et al., *Cancer Disparities and Black American Representation in Clinical Trials Leading to the Approval of Oral Chemotherapy Drugs in the United States Between 2009 and 2019.* JCO Oncol Pract, 2021. **17**(5): p. e623-e628.
5. Varma, T., et al., *Reporting of Study Participant Demographic Characteristics and Demographic Representation in Premarketing and Postmarketing Studies of Novel Cancer Therapeutics.* JAMA Netw Open, 2021. **4**(4): p. e217063.
6. Grant, S.R., et al., *Racial and Ethnic Disparities Among Participants in US-Based Phase 3 Randomized Cancer Clinical Trials.* JNCI Cancer Spectr, 2020. **4**(5): p. pkaa060.
7. Hodges, T.R., et al., *Impact of race on care, readmissions, and survival for patients with glioblastoma: an analysis of the National Cancer Database.* Neurooncol Adv, 2021. **3**(1): p. vdab040.
8. Yekedüz, E., et al., *Assessing population diversity in phase III trials of cancer drugs supporting Food and Drug Administration approval in solid tumors.* Int J Cancer, 2021. **149**(7): p. 1455-1462.
9. Phan, V.H., et al., *Ethnic differences in drug metabolism and toxicity from chemotherapy.* Expert Opin Drug Metab Toxicol, 2009. **5**(3): p. 243-57.
10. Labriola, M. and D.J. George, *Differences in Toxicity and Outcomes in Clinical Trial Participants From Minority Populations.* American Society of Clinical Oncology Educational Book, 2021(41): p. e128-e132.
11. Mauvais-Jarvis, F., et al., *Sex and gender: modifiers of health, disease, and medicine.* The Lancet, 2020. **396**(10250): p. 565-582.
12. Özdemir, B.C., et al., *Sex Differences in Efficacy and Toxicity of Systemic Treatments: An Undervalued Issue in the Era of Precision Oncology.* Journal of Clinical Oncology, 2018. **36**(26): p. 2680-2683.

© 2023 Hopkins AM et al. *JAMA Oncology*.

eAppendix. Raw Data

Data Sponsor	NCT number	Drug
AstraZeneca	NCT02000622	Olaparib (Lynparza)
AstraZeneca	NCT00753545	Olaparib (Lynparza)
AstraZeneca	NCT01078662	Olaparib (Lynparza)
AstraZeneca	NCT01874353	Olaparib (Lynparza)
AstraZeneca	NCT02296125	Osimertinib (Tagrisso)
AstraZeneca	NCT02151981	Osimertinib (Tagrisso)
Bayer	NCT01774344	Regorafenib (Stivarga)
Bayer	NCT02200614	Darolutamide (Nubeqa)
Boehringer Ingelheim	NCT00525148	Afatinib (Gilotrif)
Boehringer Ingelheim	NCT01121393	Afatinib (Gilotrif)
Boehringer Ingelheim	NCT00949650	Afatinib (Gilotrif)
Boehringer Ingelheim	NCT01523587	Afatinib (Gilotrif)
Daiichi Sankyo	NCT02371369	Pexidartinib (Turalio)
Daiichi Sankyo	NCT03248492	Trastuzumab deruxtecan (Enhertu)
Eli Lilly and Company	NCT01170663	Ramucirumab (Cyramza)
Eli Lilly and Company	NCT01168973	Ramucirumab (Cyramza)
Eli Lilly and Company	NCT02246621	Abemaciclib (Verzenio)
Eli Lilly and Company	NCT00917384	Ramucirumab (Cyramza)
Eli Lilly and Company	NCT01183780	Ramucirumab (Cyramza)
Eli Lilly and Company	NCT00982111	Necitumumab (Portrazza)
Eli Lilly and Company	NCT00981058	Necitumumab (Portrazza)
Eli Lilly and Company	NCT02102490	Abemaciclib (Verzenio)
Eli Lilly and Company	NCT02107703	Abemaciclib (Verzenio)
Hoffmann-La Roche	NCT00567190	Pertuzumab (Perjeta)
Hoffmann-La Roche	NCT00976989	Pertuzumab (Perjeta)
Hoffmann-La Roche	NCT00545688	Pertuzumab (Perjeta)
Hoffmann-La Roche	NCT01358877	Pertuzumab (Perjeta)
Hoffmann-La Roche	NCT01772472	Ado-trastuzumab emtansine (Kadcyla)
Hoffmann-La Roche	NCT02367781	Atezolizumab (Tecentriq)
Hoffmann-La Roche	NCT02075840	Alectinib (Alecensa)
Hoffmann-La Roche	NCT02366143	Atezolizumab (Tecentriq)
Hoffmann-La Roche	NCT01689519	Cobimetinib (Cotellic)
Hoffmann-La Roche	NCT02951767	Atezolizumab (Tecentriq)
Hoffmann-La Roche	NCT02763579	Atezolizumab (Tecentriq)
Hoffmann-La Roche	NCT02008227	Atezolizumab (Tecentriq)
Hoffmann-La Roche	NCT00829166	Ado-trastuzumab emtansine (Kadcyla)
Hoffmann-La Roche	NCT03434379	Atezolizumab (Tecentriq)
Hoffmann-La Roche	NCT02908672	Atezolizumab (Tecentriq)
Hoffmann-La Roche	NCT00833417	Vismodegib (Erivedge)
Hoffmann-La Roche	NCT01006980	Vemurafenib (Zelboraf)
Hoffmann-La Roche	NCT00949702	Vemurafenib (Zelboraf)
Hoffmann-La Roche	NCT01378975	Vemurafenib (Zelboraf)
Janssen Research & Development, LLC	NCT01715285	Abiraterone (Zytiga)
Janssen Research & Development, LLC	NCT00887198	Abiraterone (Zytiga)
Janssen Research & Development, LLC	NCT00638690	Abiraterone (Zytiga)
Janssen Research & Development, LLC	NCT01343277	Trabectedin (Yondelis)
Merck Group / EMD Serono	NCT02155647	Avelumab (Bavencio)
Novartis	NCT01597908	Dabrafenib (Tafinlar)

© 2023 Hopkins AM et al. *JAMA Oncology*.

Novartis	NCT01266967	Dabrafenib (Tafinlar)
Novartis	NCT01227889	Dabrafenib (Tafinlar)
Novartis	NCT01584648	Trametinib (Mekinist)
Novartis	NCT01336634	Trametinib (Mekinist)
Novartis	NCT02039947	Trametinib (Mekinist)
Pfizer	NCT01740427	Palbociclib (Ibrance)
Pfizer	NCT01970865	Lorlatinib (Lorbrena)
Pfizer	NCT02928224	Encorafenib (Braftovi)
Pfizer	NCT01154140	Crizotinib (Xalkori)
Pfizer	NCT01942135	Palbociclib (Ibrance)
Pfizer	NCT00932893	Crizotinib (Xalkori)
Pfizer	NCT00678392	Axitinib (Inlyta)
Pfizer	NCT01909453	Binimetinib (Mektovi)
Pfizer	NCT01774721	Dacomitinib (Vizimpro)
Pfizer	NCT02603432	Avelumab (Bavencio)
Pfizer	NCT01945775	Talazoparib (Talzenna)
Puma Biotechnology	NCT00878709	Neratinib (Nerlynx)
Puma Biotechnology	NCT01808573	Neratinib (Nerlynx)
Sanofi	NCT00410761	Vandetanib (Caprelsa)
Sanofi	NCT00561470	Ziv-aflibercept (Zaltrap)
Takeda	NCT02737501	Brigatinib (Alunbrig)
Takeda	NCT02094573	Brigatinib (Alunbrig)

© 2023 Hopkins AM et al. *JAMA Oncology*.

Drug Class	Condition Class	Data Sharing Platform
Non-Cytotoxic Targeted Drugs	Breast Cancer	Vivli
Non-Cytotoxic Targeted Drugs	Ovarian Cancer	Vivli
Non-Cytotoxic Targeted Drugs	Solid	Vivli
Non-Cytotoxic Targeted Drugs	Ovarian Cancer	Vivli
Non-Cytotoxic Targeted Drugs	Lung Cancer	Vivli
Non-Cytotoxic Targeted Drugs	Solid	Vivli
Non-Cytotoxic Targeted Drugs	Liver Cancer	Vivli
Hormonal Drugs	Prostate Cancer	Vivli
Non-Cytotoxic Targeted Drugs	Lung Cancer	Vivli
Non-Cytotoxic Targeted Drugs	Solid	Vivli
Non-Cytotoxic Targeted Drugs	Solid	Vivli
Non-Cytotoxic Targeted Drugs	Lung Cancer	Vivli
Non-Cytotoxic Targeted Drugs	Solid	Vivli
Antibody-Drug Conjugates	Breast Cancer	Vivli
Non-Cytotoxic Targeted Drugs	Stomach Cancer	Vivli
Non-Cytotoxic Targeted Drugs	Lung Cancer	Vivli
Non-Cytotoxic Targeted Drugs	Breast Cancer	Vivli
Non-Cytotoxic Targeted Drugs	Solid	Vivli
Non-Cytotoxic Targeted Drugs	Colon and Rectal Cancer	Vivli
Non-Cytotoxic Targeted Drugs	Lung Cancer	Vivli
Non-Cytotoxic Targeted Drugs	Lung Cancer	Vivli
Non-Cytotoxic Targeted Drugs	Breast Cancer	Vivli
Non-Cytotoxic Targeted Drugs	Breast Cancer	Vivli
Non-Cytotoxic Targeted Drugs	Breast Cancer	Vivli
Non-Cytotoxic Targeted Drugs	Breast Cancer	Vivli
Non-Cytotoxic Targeted Drugs	Breast Cancer	Vivli
Non-Cytotoxic Targeted Drugs	Breast Cancer	Vivli
Antibody-Drug Conjugates	Breast Cancer	Vivli
Immunomodulatory Drugs	Lung Cancer	Vivli
Non-Cytotoxic Targeted Drugs	Lung Cancer	Vivli
Immunomodulatory Drugs	Lung Cancer	Vivli
Non-Cytotoxic Targeted Drugs	Melanoma	Vivli
Immunomodulatory Drugs	Bladder Cancer	Vivli
Immunomodulatory Drugs	Lung Cancer	Vivli
Immunomodulatory Drugs	Lung Cancer	Vivli
Antibody-Drug Conjugates	Breast Cancer	Vivli
Immunomodulatory Drugs	Liver Cancer	Vivli
Immunomodulatory Drugs	Melanoma	Vivli
Non-Cytotoxic Targeted Drugs	Solid	Vivli
Non-Cytotoxic Targeted Drugs	Melanoma	Vivli
Non-Cytotoxic Targeted Drugs	Melanoma	Vivli
Non-Cytotoxic Targeted Drugs	Melanoma	Vivli
Hormonal Drugs	Prostate Cancer	YODA
Hormonal Drugs	Prostate Cancer	YODA
Hormonal Drugs	Prostate Cancer	YODA
Cytotoxic Drugs	Solid	YODA
Immunomodulatory Drugs	Solid	InternalPlatform
Non-Cytotoxic Targeted Drugs	Melanoma	CSDR

© 2023 Hopkins AM et al. *JAMA Oncology*.

Non-Cytotoxic Targeted Drugs	Melanoma	CSDR
Non-Cytotoxic Targeted Drugs	Solid	CSDR
Non-Cytotoxic Targeted Drugs	Melanoma	CSDR
Non-Cytotoxic Targeted Drugs	Solid	CSDR
Non-Cytotoxic Targeted Drugs	Melanoma	CSDR
Non-Cytotoxic Targeted Drugs	Breast Cancer	Vivli
Non-Cytotoxic Targeted Drugs	Lung Cancer	Vivli
Non-Cytotoxic Targeted Drugs	Colon and Rectal Cancer	Vivli
Non-Cytotoxic Targeted Drugs	Lung Cancer	Vivli
Non-Cytotoxic Targeted Drugs	Breast Cancer	Vivli
Non-Cytotoxic Targeted Drugs	Lung Cancer	Vivli
Non-Cytotoxic Targeted Drugs	Kidney Cancer	Vivli
Non-Cytotoxic Targeted Drugs	Melanoma	Vivli
Non-Cytotoxic Targeted Drugs	Lung Cancer	Vivli
Immunomodulatory Drugs	Bladder Cancer	Vivli
Non-Cytotoxic Targeted Drugs	Breast Cancer	Vivli
Non-Cytotoxic Targeted Drugs	Breast Cancer	InternalPlatform
Non-Cytotoxic Targeted Drugs	Breast Cancer	InternalPlatform
Non-Cytotoxic Targeted Drugs	Thyroid Cancer	Vivli
Non-Cytotoxic Targeted Drugs	Colon and Rectal Cancer	Vivli
Non-Cytotoxic Targeted Drugs	Lung Cancer	Vivli
Non-Cytotoxic Targeted Drugs	Lung Cancer	Vivli

© 2023 Hopkins AM et al. *JAMA Oncology*.

Data Request Submission Date	Primary Completion Date of Trial	Trial Phase
2/10/2022	12/9/2016	Phase 3
2/10/2022	6/30/2010	Phase 2
2/10/2022	7/31/2012	Phase 2
2/10/2022	9/19/2016	Phase 3
2/10/2022	6/19/2017	Phase 3
2/10/2022	4/15/2016	Phase 3
2/9/2022	2/29/2016	Phase 3
2/9/2022	9/3/2018	Phase 3
2/10/2022	1/2/2010	Phase 2
2/10/2022	11/23/2017	Phase 3
2/10/2022	2/9/2012	Phase 3
2/10/2022	10/21/2013	Phase 3
2/10/2022	3/27/2017	Phase 3
2/10/2022	3/21/2019	Phase 2
2/10/2022	1/7/2013	Phase 3
2/10/2022	12/1/2013	Phase 3
2/10/2022	1/31/2017	Phase 3
2/10/2022	1/7/2012	Phase 3
2/10/2022	7/17/2014	Phase 3
2/10/2022	11/14/2012	Phase 3
2/10/2022	6/17/2013	Phase 3
2/10/2022	4/30/2016	Phase 2
2/10/2022	2/14/2017	Phase 3
2/10/2022	5/13/2011	Phase 3
2/10/2022	1/6/2011	Phase 2
2/10/2022	9/22/2014	Phase 2
2/10/2022	12/19/2016	Phase 3
2/10/2022	7/25/2018	Phase 3
2/10/2022	3/15/2018	Phase 3
2/10/2022	2/9/2017	Phase 3
2/10/2022	9/13/2019	Phase 3
2/10/2022	5/9/2014	Phase 3
2/10/2022	5/31/2015	Phase 2
2/10/2022	4/24/2018	Phase 3
2/10/2022	7/7/2016	Phase 3
2/10/2022	1/7/2012	Phase 3
2/10/2022	6/2/2021	Phase 3
2/10/2022	10/11/2019	Phase 3
2/10/2022	1/11/2010	Phase 2
2/10/2022	1/12/2010	Phase 3
2/10/2022	9/27/2010	Phase 2
2/10/2022	1/7/2015	Phase 2
2/9/2022	10/31/2016	Phase 3
2/9/2022	3/31/2014	Phase 3
2/9/2022	1/8/2010	Phase 3
2/9/2022	1/1/2015	Phase 3
2/9/2022	5/2/2019	Phase 2
2/9/2022	4/17/2014	Phase 3

© 2023 Hopkins AM et al. *JAMA Oncology*.

2/9/2022	1/11/2011	Phase 2
2/9/2022	12/19/2011	Phase 3
2/9/2022	8/26/2013	Phase 3
2/9/2022	10/1/2015	Phase 2
2/9/2022	5/12/2017	Phase 2
2/10/2022	2/26/2016	Phase 3
2/10/2022	3/15/2017	Phase 2
2/10/2022	2/11/2019	Phase 3
2/10/2022	11/30/2013	Phase 3
2/10/2022	12/5/2014	Phase 3
2/10/2022	1/3/2012	Phase 3
2/10/2022	8/31/2010	Phase 3
2/10/2022	11/9/2016	Phase 3
2/10/2022	1/7/2016	Phase 3
2/10/2022	10/21/2019	Phase 3
2/10/2022	9/15/2017	Phase 3
2/9/2022	8/21/2014	Phase 3
2/9/2022	9/28/2018	Phase 3
2/10/2022	1/7/2009	Phase 3
2/10/2022	1/2/2011	Phase 3
2/10/2022	7/28/2020	Phase 3
2/10/2022	2/29/2016	Phase 2

© 2023 Hopkins AM et al. *JAMA Oncology*.

Trial IPD sharec	Date of IPD receipt	Days to IPD receipt
YES	12/3/2022	296
YES	11/30/2022	293
YES	12/19/2022	312
YES	11/18/2022	281
YES	1/28/2023	352
YES	11/18/2022	281
YES	12/14/2022	308
YES	12/14/2022	308
YES	6/13/2022	123
YES	6/13/2022	123
YES	6/13/2022	123
YES	6/13/2022	123
YES	6/9/2022	119
YES	6/9/2022	119
YES	6/7/2022	117
YES	6/7/2022	117
YES	6/7/2022	117
YES	6/22/2022	132
YES	6/7/2022	117
YES	6/22/2022	132
YES	6/23/2022	133
YES	6/7/2022	117
YES	6/7/2022	117
YES	6/13/2022	123
YES	6/22/2022	132
YES	6/9/2022	119
YES	6/13/2022	123
YES	6/10/2022	120
YES	6/17/2022	127
YES	6/17/2022	127
YES	6/17/2022	127
YES	6/13/2022	123
YES	6/10/2022	120
YES	6/13/2022	123
YES	6/13/2022	123
YES	6/10/2022	120
YES	6/7/2022	117
YES	6/10/2022	120
YES	6/10/2022	120
YES	6/13/2022	123
YES	6/10/2022	120
YES	6/13/2022	123
YES	6/8/2022	119
YES	6/8/2022	119
YES	6/8/2022	119
YES	6/8/2022	119
YES	7/18/2022	159
YES	8/20/2022	192

© 2023 Hopkins AM et al. *JAMA Oncology*.

YES	8/20/2022	192
YES	8/20/2022	192
YES	8/20/2022	192
YES	8/20/2022	192
YES	8/20/2022	192
YES	6/10/2022	120
YES	6/10/2022	120
YES	7/19/2022	159
YES	7/19/2022	159
YES	6/10/2022	120
YES	6/10/2022	120
YES	6/10/2022	120
YES	7/6/2022	146
YES	6/10/2022	120
YES	6/10/2022	120
YES	7/6/2022	146
YES	9/16/2022	219
YES	9/16/2022	219
YES	7/27/2022	167
YES	7/18/2022	158
YES	6/7/2022	117
YES	6/10/2022	120

© 2023 Hopkins AM et al. *JAMA Oncology*.

Provision of key IPD variables						
**Adverse event**	**Survival**	**PFS/DFS**	**Race**	**Sex**	**Age**	**Weight**
Partially redacted	YES	YES	Partially redacted	YES	Partially redact	NO
Partially redacted	YES	YES	YES	YES	Partially redact	NO
Partially redacted	YES	YES	YES	YES	Partially redact	NO
Partially redacted	YES	YES	YES	YES	Partially redact	NO
Partially redacted	YES	NO	YES	YES	Partially redact	NO
Partially redacted	YES	NO	Partially redacted	YES	Partially redact	NO
YES	YES	YES	YES	YES	YES	YES
YES	YES	YES	YES	YES	YES	YES
YES	YES	YES	YES	YES	YES	YES
YES	YES	YES	YES	YES	YES	YES
YES	YES	YES	YES	YES	YES	YES
YES	YES	YES	YES	YES	YES	YES
Partially redacted	NA	NA	NO	YES	Partially redact	NO
Partially redacted	NO	NO	NO	YES	Partially redact	NO
YES	YES	YES	YES	YES	YES	YES
YES	YES	YES	YES	YES	YES	YES
YES	NA	YES	YES	YES	YES	YES
YES	YES	YES	YES	YES	YES	YES
YES	YES	YES	YES	YES	YES	YES
YES	YES	YES	YES	YES	YES	YES
YES	YES	YES	YES	YES	YES	YES
YES	YES	YES	YES	YES	YES	YES
YES	YES	YES	YES	YES	YES	YES
YES	YES	YES	YES	YES	YES	YES
YES	YES	YES	YES	YES	YES	YES
YES	NA	YES	YES	YES	YES	YES
YES	YES	YES	YES	YES	YES	YES
YES	YES	YES	YES	YES	YES	YES
YES	YES	YES	YES	YES	YES	YES
YES	YES	YES	YES	YES	YES	YES
YES	YES	YES	YES	YES	YES	YES
YES	YES	YES	YES	YES	YES	YES
YES	YES	YES	YES	YES	YES	YES
YES	YES	YES	YES	YES	YES	YES
YES	YES	YES	YES	YES	YES	YES
YES	YES	YES	YES	YES	YES	YES
YES	YES	YES	YES	YES	YES	YES
YES	YES	YES	YES	YES	YES	YES
YES	YES	YES	YES	YES	YES	YES
YES	YES	YES	YES	YES	YES	YES
YES	YES	YES	YES	YES	YES	YES
YES	YES	YES	YES	YES	YES	YES
YES	YES	YES	YES	YES	Partially redact	YES
YES	YES	YES	YES	YES	YES	YES
YES	YES	YES	YES	YES	YES	YES
YES	YES	YES	YES	YES	YES	YES
YES	YES	YES	Partially redacted	YES	Partially redact	YES
YES	YES	NO	YES	YES	Partially redact	YES

© 2023 Hopkins AM et al. *JAMA Oncology*.

YES	YES	YES	NO	YES	Partially redact	YES
YES	YES	YES	NO	YES	Partially redact	YES
YES	YES	YES	YES	YES	Partially redact	YES
YES	YES	YES	YES	YES	Partially redact	YES
YES	YES	YES	YES	YES	Partially redact	YES
YES	NA	YES	YES	YES	YES	YES
YES	NO	YES	YES	YES	YES	NO
Partially redacted	YES	YES	YES	YES	YES	YES
YES	NO	YES	YES	YES	YES	YES
YES	YES	YES	YES	YES	YES	YES
YES	YES	YES	YES	YES	YES	YES
YES	YES	YES	YES	YES	YES	YES
YES	NA	YES	YES	YES	YES	YES
Partially redacted	YES	YES	YES	YES	YES	YES
Partially redacted	YES	YES	YES	YES	YES	YES
Partially redacted	YES	YES	YES	YES	YES	YES
YES	NO	NO	YES	YES	YES	YES
YES	NO	NO	YES	NO	YES	YES
YES	NO	YES	Partially redacted	YES	Partially redact	NO
YES	YES	YES	Partially redacted	YES	YES	YES
YES	YES	YES	YES	YES	YES	YES
YES	YES	YES	NO	YES	Partially redact	YES

© 2023 Hopkins AM et al. *JAMA Oncology*.

			Supporting documentation prov
Performance status	CSR	Data dictionary	Data derivation specifications
YES	YES	YES	NO
YES	YES	YES	NO
YES	YES	YES	NO
YES	YES	YES	NO
YES	YES	YES	NO
YES	YES	YES	NO
YES	NO	YES	YES
YES	NO	YES	YES
YES	YES	YES	YES
YES	YES	YES	YES
YES	YES	YES	YES
YES	YES	YES	YES
NA	NO	YES	NO
NO	NO	YES	NO
YES	YES	YES	YES
YES	YES	YES	YES
YES	NO	YES	YES
YES	YES	YES	YES
YES	YES	YES	YES
YES	YES	YES	YES
YES	YES	YES	YES
YES	YES	YES	YES
YES	NO	YES	YES
YES	YES	YES	NO
YES	YES	YES	YES
YES	YES	Partially	Partially
YES	YES	YES	YES
YES	YES	YES	YES
YES	YES	YES	YES
YES	YES	YES	YES
YES	YES	YES	YES
YES	YES	YES	NO
YES	YES	YES	YES
YES	YES	YES	YES
YES	YES	YES	YES
YES	YES	Partially	Partially
YES	YES	YES	YES
YES	YES	YES	YES
YES	YES	YES	YES
YES	YES	YES	NO
YES	YES	YES	YES
YES	YES	YES	YES
YES	NO	NO	NO
YES	YES	YES	YES
YES	YES	YES	NO
YES	YES	YES	YES
YES	NO	YES	NO
YES	YES	YES	YES

© 2023 Hopkins AM et al. *JAMA Oncology*.

YES	YES	NO	NO
YES	YES	YES	YES
YES	YES	YES	YES
YES	NO	YES	YES
YES	YES	YES	YES
YES	NO	YES	YES
NO	NO	YES	YES
YES	NO	YES	YES
YES	NO	YES	YES
YES	NO	YES	YES
YES	NO	YES	YES
YES	NO	YES	YES
YES	NO	YES	YES
YES	NO	YES	NO
YES	NO	YES	YES
YES	NO	YES	YES
YES	YES	YES	YES
YES	YES	YES	YES
YES	YES	YES	NO
YES	YES	YES	YES
YES	YES	YES	NO
YES	YES	YES	NO

© 2023 Hopkins AM et al. *JAMA Oncology*.

ision

Anonymisation orientation file	Reason provided for not sharing IPD
YES	
YES	
YES	
YES	
YES	
YES	
YES	
YES	
YES	
YES	
YES	
YES	
YES	
YES	
NO	
NO	
NO	
NO	
NO	
NO	
NO	
NO	
NO	
YES	
YES	
YES	
YES	
YES	
YES	
YES	
YES	
YES	
YES	
YES	
YES	
YES	
YES	
YES	
YES	
YES	
YES	
YES	
YES	
YES	
NO	
YES	
YES	
NO	

© 2023 Hopkins AM et al. *JAMA Oncology*.

NO
NO
NO
NO
NO
YES
YES
YES
YES
YES
YES
YES
YES
YES
YES
YES
NO
NO
YES
YES
NO
NO

© 2023 Hopkins AM et al. *JAMA Oncology*.

IPD avaliable on Project DataSphere
NO
NO
NO
NO
NO
NO
NO
NO
NO
NO
NO
NO
NO
NO
NO
NO
NO
NO
NO
NO
YES
NO
NO
NO
NO
NO
NO
NO
NO
NO
NO
NO
NO
NO
NO
NO
NO
NO
NO
NO
NO
NO
NO
NO
NO
NO
NO
NO

© 2023 Hopkins AM et al. *JAMA Oncology*.

NO
NO
NO
NO
NO
NO
NO
NO
NO
NO
NO
NO
NO
NO
NO
NO
NO
NO
NO
YES
NO
NO

© 2023 Hopkins AM et al. *JAMA Oncology*.
