# Supplementary material for: Heterogeneity and Utility of Pharmaceutical Company Sharing of Individual-Participant Data Packages
Source: JAMA Oncol. 2023 Oct 5;9(12):1621–6. doi: 10.1001/jamaoncol.2023.3996 (PMC10557028; doi:10.1001/jamaoncol.2023.3996)
Supplement: Supplement 2. — Data Sharing Statement [file jamaoncol-e233996-s002.pdf]

Data Sharing Statement

Hopkins. Heterogeneity and Utility of Pharmaceutical Company Sharing of Individual-Participant Data Packages. *JAMA Oncol*. Published October 05, 2023. doi:10.1001/jamaoncol.2023.3996

Data
Data available: Yes
Data types: Other (please specify)
Additional Information: Supplementary Files contain the research proposal and raw data.
How to access data: They will be uploaded alongside the manuscript
When available: With publication

Supporting Documents
Document types: None

Additional Information
Who can access the data: Anyone requesting the data
Types of analyses: For any purpose or for a specified purpose
Mechanisms of data availability: NA
